# Biochar with wheat straw and farmyard manure modulate microbial activity by inducing qualitative and quantitative changes in soil organic carbon in saline, sodic and saline-sodic soils

**DOI:** 10.7717/peerj.21100

**Published:** 2026-05-21

**Authors:** Samar Fatima, Muhammad Riaz, Ivica Djalovic, P.V. Vara Prasad

**Affiliations:** 1 Department of Environmental Sciences, Government College University Faisalabad, Faisalabad, Pakistan; 2 Institute of Field and Vegetable Crops, National Institute of the Republic of Serbia, Novi Sad, Serbia; 3 Department of Agronomy, Kansas State University, Manhattan, KS, United States of America

**Keywords:** Biochar, Salinity, Soil organic carbon, Microbial activity, Decomposition, Farmyard manure, Extracellular soil enzymes

## Abstract

Soil salinity is a global soil degradation issue and poses significant threats to soil quality and food security. Saline soils are inherently poor in soil organic matter, microbial activity and plant available nutrients. We investigated the effects of biochar (BC), wheat straw (WS) and farmyard manure (FYM) on microbial activity, qualitative and quantitative soil organic carbon (SOC) dynamics, activities of extracellular enzymes and nutrient availability in saline, sodic and saline-sodic soils. The incubation study followed completely randomized experimental design consisting of six treatments: (1) unamended control; (2) BC, derived from corncob biowaste (2%, w/w basis); (3) WS(2% w/w); (4) FYM (2%, w/w); (5) BC+WS (each 1%, w/w); and, (6) BC+FYM (each 1%, w/w). Biochar significantly reduced decomposition of WS and FYM in saline soils. Biochar with and without WS and FYM significantly reduced soil pH and electrical conductivity (EC) in all saline soils. Biochar promoted microbial activity by significantly increasing microbial biomass carbon (C), nitrogen (N) and phosphorus (P) and nutrient pools in all saline soils but much higher in sodic soil. Soil microbial biomass and mineral nutrients corresponded positively to soil dehydrogenase, β-glucosidase, urease and alkaline phosphomonesterase enzymatic activities. Biochar also significantly enhanced extractable (cold- and hot-water) WEOC and total SOC contents when applied with WS and FYM. Biochar with WS and FYM significantly reduced dissolved organic C (DOC) contents suggesting higher C retention resulted from changes in SOC quality as indicated from the specific ultraviolet absorbance (SUVA) and E2/E3 ratio. Biochar reduced C mineralization of fresh organic matter (WS and FYM), retained more C, improved soil biochemical functions and altered SOC quality. We conclude that BC along with other fresh organic matter inputs can improve soil organic matter cycling and biochemical functions to restore soil quality and health in saline soils.

## Introduction

Soil salinity has become a significant source of land degradation (Rath & Rousk, 2015; Singh, 2022), affecting nearly one-third of irrigated land globally (FAO & ITPS, 2015). Soil salinity is catalyzing decline in agricultural production, provision of ecosystem services, soil quality and economic wellbeing (Amundson et al., 2015; Keesstra et al., 2016). Salt affected soils are characterized by higher concentrations of certain ions including sodium, calcium, magnesium, chlorides, sulfates, carbonates and bicarbonates (Manchanda & Garg, 2008). However, depending upon sodium and salt concentrations, these are classified as saline, sodic and saline-sodic soils. Saline and sodic soils constitute around 40 and 60% of global share of salt affected soils, respectively, and are usually found in arid and semiarid regions (Qadir, Ghafoor & Murtaza, 2000). Currently, 935 million ha land comes under salt affected soils causing an estimated loss of 27.3 billion US dollars (Qadir et al., 2014; Ivushkin et al., 2019). Soil salinity is expected to increase due to rapidly changing climate (Hassani, Azapagic & Shokri, 2021). However, in Pakistan, salinity has affected around 20% of total cultivated land area (Aslam, 2016).

Salt affected alkaline soils tend to have low soil organic matter contents because of limited fresh organic matter inputs, deteriorated soil physicochemical properties and decreased water availability (Setia et al., 2010; Amini et al., 2016). Accelerated organic matter decomposition due to hot climate is another compelling factor for low soil organic carbon (SOC) in arid and semiarid region soils (Souri, Naiji & Kianmehr, 2019). Under high salinity, there may be an increased desorption of soil organic matter resulting in increased dissolved organic carbon (DOC) in soil solution that tends to get transported and removed through irrigation, leaching and ground water movement (Setia, Rengasamy & Marschner, 2013; Singh, Singh & Singh, 2023).

Salinity causes adverse effects on soil functions (*e.g.*, soil respiration, soil microbial biomass and enzyme activity) that are involved in organic matter turnover and nutrient cycling (Rath & Rousk, 2015; Leogrande & Vitti, 2019). Salinity can affect soil respiration by altering microbial community structure and activities (Rath & Rousk, 2015). Soil respiration, microbial biomass and enzymatic activities usually decrease with increasing salinity due to low osmotic potential and inability of microbes to adapt to high salt concentrations (Chowdhury, Burns & Marschner, 2011; Morrissey et al., 2014). However, the inhibited microbial activity is attributed to negative effects of salinity and low organic matter inputs due to limited plant growth in saline soils (Rath & Rousk, 2015).

Application of organic amendments into soil can help alleviate the negative effects of salinity by improving physicochemical and biological properties of soil (Chahal, Choudhary & Mavi, 2017; Leogrande & Vitti, 2019). The available C from organic matter is used by microbes to produce osmolytes to counteract osmotic stress (Tang et al., 2025). Furthermore, microbial decomposition of crop residues results in provision of essential nutrients in soil, particularly in case of low external inputs. Organic amendments also improve soil aggregation, increases cation exchange capacity (Schulz & Glaser, 2012), microbial activity and ultimately soil fertility (Ding et al., 2020). However, the benefits of such organic amendments are often short-lived, since decomposition rates are high and mineralization of the added organic matter happens within a time of only a few weeks to months (Chaganti, Crohn & Šimůnek, 2015).

Biochar (BC) is a carbon rich material obtained through pyrolysis of organic waste below 700 °C in an oxygen limited environment (Xiao et al., 2018). Biochar has shown potential in reclaiming degraded soils (Yuan et al., 2018), improving soil quality by increasing cation exchange capacity (Kookana et al., 2011), improving soil structure (increased soil porosity and water retention) (Jones et al., 2012), enhancing nutrient retention (Clough et al., 2013), benefitting soil microbial biomass and composition (Lehmann et al., 2015) and reducing greenhouse gas emissions (Fidel, Parkin & Laird, 2019; Ding et al., 2020; Wei et al., 2021). BC addition to saline soil has shown improved soil quality and plant growth in previous studies (Ali et al., 2017; Huang et al., 2019; Rekaby et al., 2020; Wu et al., 2024). Soil organic C contents in arid and semi-arid soils are critically low, and BC amendments have shown significant potential to increase SOC contents in these agroecosystems (Gross, Glaser & Bromm, 2021; Thapa et al., 2023). Moreover, a meta-analysis by Su et al. (2024) revealed a promising role of BC to remediate salt-affected soils and, thus enhancing soil properties and plant productivity. However, the extent of these benefits depends on the characteristics of BC (pyrolysis temperature, duration and feedstock nature) and soil type (Xiao et al., 2018), suggesting the higher variations in results reported by different studies (Tomczyk, Sokołowska & Boguta, 2020; Cai et al., 2021).

Combined application of BC and organic amendments like manure results in increased concentration of Ca^2+^ in soil solution which subsequently removes Na^+^ from exchange sites (Srivastava et al., 2016; Yaduvanshi, 2017). Moreover, improved soil structure and aggregate stability due to added organic amendments also enhance permeability which facilitates Na^+^ removal from soil profile *via* leaching (Andrade Foronda, 2022). The combined application of BC and fresh organic matter can be more favorable for soils due to simultaneous functioning of synergistic mechanisms between a recalcitrant and a labile C source (Fang et al., 2019). The combined application of BC and fresh organic matter is still at an initial stage due to:

• Contradictory nature of both biochar and data available on effects of BC on nutrient release (Purakayastha et al., 2019); and,
• The magnitude and intensity of interactive effects between BC and fresh organic matter mainly depend on their quality (Majumder et al., 2019).

Thus, the combined application of BC with organic and inorganic fertilizer could enhance beneficial effects of BC (Yu et al., 2019). Many studies have suggested co-application of BC with other nutrient rich organic amendments for its reaping maximum multifunctional benefits (*e.g.*, Arif et al., 2017; Fu et al., 2023).

It was hypothesized that BC would induce qualitative and quantitative changes in SOC resulting from decomposition of fresh organic matter (wheat straw (WS) and farmyard manure (FYM)), and thus, influence microbial activity and biomass, activities of extra-cellular soil enzymes and soil nitrogen (N) and phosphorus (P) dynamics in low C saline soils; however, these interactions would depend on the degree of salinity. Therefore, the objectives of this incubation study were to assess the effects of BC, with and without WS and FYM, on microbial activity and biomass, activities of dehydrogenase, β-glucosidase, urease and alkaline phosphomonoesterase enzymes, C pools and NP contents in saline soils.

## Materials & Methods

### Study sites and sampling

The saline, sodic and saline sodic soils for incubation study were taken from Mianwali (32.5839°N, 71.5370°E), Sargodha (32.0740°N, 72.6861°E) and Sahiwal (30.6682°N, 73.1114°E), respectively. Soils were sampled from the depth of 0–15 cm with an augur. Any live/dead vegetation and stones were removed from soil samples in the field and they were air-dried before sieving through a 2.0 mm mesh. The subsequent soils were then mixed thoroughly to obtain composite samples of homogenous and uniform nature. Soil samples were stored in plastic bags and kept in the dark until used in experiments.

### Collection of biowaste materials and biochar production

Corncobs, WS and FYM were collected from the research-farm of the University of Agriculture Faisalabad, Pakistan (31.4294°N, 73.0750°E). Corncob, WS and FYM were sun-dried for two weeks, crushed and passed through a 2.0 mm sieve, and stored in the dark. BC was prepared from corncob residues following the method of Sánchez et al. (2009). Pyrolysis of biomass was carried out in a furnace having a Pyrex flask of 2-L volume that was connected to a U-shaped glass tube (1-ft height and 2.5-ft basal length) at a 60° position to function as an exhaust for vapors produced during the procedure. The Pyrex flask was linked to a glass tube with temperature resistant silicon glue to keep reaction chamber oxygen free. During pyrolysis, temperature increased at a rate of 7–10 °C min^−1^ until 450 °C and heating at this temperature was sustained for 30 mins. Distilled water was sprinkled into the reaction vessel to extinguish pyrolysis, and biochar was dried in an oven at 50 °C for 24 h.

### Preliminary analysis of soil, wheat straw, farmyard manure and biochar

Soil pH and electrical conductivity (EC) were quantified in soil solution at 1:5 (soil to solvent ratio) with the help of pre-calibrated pH and EC meters (HANNA-210; HANNA-HI 8733), respectively. However, pH and EC of WS, FYM and BC were measured using 1:10 ratio following the similar protocol.

Total C and N contents of WS, FYM and BC were determined on a CNS analyzer (Elementar Vario Macro, Germany). Samples were oven-dried at 70 °C for 24 h followed by grinding with a ball-mill to form homogenous powder. These samples were then analyzed for total C and N contents following an automated procedure and calibrating the analyzer with glutamic acid. Soil particle size distribution and texture was measured using the Bouyoucos hydrometer method (Bouyoucos, 1962).

### Experimental design, treatments and incubation

Three subsamples of about 1,000 grams were transferred from each composite-sample of saline, sodic and saline sodic soil into resealable plastic bags. Distilled water was added into these samples through continuous mixing to bring their moisture contents equivalent to 50% water holding capacity. Samples were placed in an incubator at 20 ± 1 °C for 14-days, with occasional light mixing to rejuvenate microbial activity (Nguyen & Marschner, 2014). For incubation experiments, microcosms were constructed from PVC pipes (three cm diameter, 10 cm height/length) with a nylon mesh at the base.

The two-factor completely randomized experimental design consisted of six treatments, replicated three times: control (soil without any amendment), BC (2% biochar to soil, w/w basis), WS (2% wheat straw-soil, w/w basis), FYM (farmyard manure-soil at 2% rate, w/w basis), BC + WS (1% each both biochar plus wheat straw, w/w basis), BC + FYM (1% each both biochar plus farmyard manure, w/w basis), and three saline soils: saline, sodic and saline-sodic. The rates of 1% and 2% application for each amendment were equivalent to 22.5 and 45 tons per hectare, respectively. under the assumption that they were mixed into the first 15 cm of soil having bulk density of 1.6 Mg m^−3^. The application rate was determined based on prior research indicating that a 45 tons per hectare rate of BC could reduce the decomposition of both added and native organic C while improved microbial C utilization efficiency (Riaz et al., 2017).

WS, FYM and BC without the addition of mineral nutrients were mixed homogeneously with 80 g of pre-conditioned soil and soil sample was moistened to 60% water holding capacity by adding distilled water. After treatment application, soil samples were re-packed into microcosms with gentle shaking to the depth equivalent to 1.6 Mg m^−3^ bulk density. The microcosms were transferred into 1.5 L mason jars along with plastic vials containing 20 mL 0.1N NaOH to capture CO_2_ released from soil microbial activity. Smaller plastic vials of 10 mL distilled water were also placed in mason jars to sustain internal humidity and moist conditions during incubation. Soil samples were then incubated at 20 ± 1 °C. During incubation period, NaOH vials were replaced at regular intervals and, each microcosm was weighed to maintain soil moisture level by replenishing the lost soil moisture with distilled water.

### Measurement of soil respiration

Soil C mineralization was quantified by measuring CO_2_ released from the soil. After each incubation interval, NaOH traps were treated with two mL 0.5M BaCl_2_ to precipitate excess CO_2_ and were back titrated against 0.5M HCl using phenolphthalein indicator. Amount of CO_2_ was calculated using following formula:

(1) \begin{eqnarray*}{\mathrm{CO}}_{2}-\mathrm{C}~({\mathrm{mg~ CO}}_{2}-{\mathrm{Cg}}^{-1}~\mathrm{soil})=(C-S)\times 2.2\times 100/\mathrm{SW}\times \%~\mathrm{dm}\end{eqnarray*}

where,

C: HCl volume for blank

S: HCl volume for sample

2.2: conversion-factor (one mL 0.1 M HCl is equivalent to 2.2 mg CO_2_)

SW: soil-weight (g)

100/% dm: factor for dry matter calculations

CO_2_ efflux was calculated by adding up the individual CO_2_ values for each sample measured during incubation.

### Post experimental soil analysis

After the 60-days incubation period, soil samples were removed from microcosms, transferred into resealable plastic bags and immediately stored at 4 °C in a refrigerator until further analysis. Soil moisture content was measured following the gravimetric-method by drying soil samples in a fan-forced oven at 105 °C overnight. Soil pH and EC were determined using moist samples, while oven-dried samples were used for SOC analysis, following the procedures described earlier.

Both the cold- and hot-water extractable organic C (WEOC) contents were determined following the procedure described by Ghani, Dexter & Perrott (2003). Five-gram moist soil samples were transferred into 50-mL plastic vials, to which 25 mL distilled water was added. The suspension was shaken on a horizontal shaker at 150 rpm for 90 min and then centrifuged at 5,000 rpm for 10 min. The resulting supernatant was filtered through a Whatman#42 filter paper and stored at 4 °C until analysis. After extractions for cold-WEOC, 25 mL distilled water was added to each plastic vial, and they were placed in a constant temperature water bath at 70 °C for over-night. Extracts from these samples were collected following the protocol similar to cold-WEOC. Dissolved organic C (DOC) was collected by leaching five g samples with 25 mL distilled water on Whatman#42 filter papers. Total organic C contents in the extracts and leachate samples were determined using the Walkley and Black method. Absorbance of the leachate samples collected for DOC was measured at 254 and 365 nm on a double beam spectrophotometer (Dynamica Halo DB-20 Series, Livingston, UK) to calculate specific ultraviolet absorbance and E2/E3 ratios (Xue et al., 2022).

Microbial biomass C (MBC), microbial biomass N (MBN), and microbial biomass P (MBP) were assessed using the chloroform fumigation extraction method (Brookes et al., 1985). The first set of 20-g duplicate soil samples was fumigated with ethanol-free chloroform for 24 h in a desiccator. Simultaneously, the second set of non-fumigated soil samples was divided into two 10-gram subsets. One subset was extracted with 40 mL of 0.5 M K_2_SO_4_, and the other with 0.5 M NaHCO_3_ by shaking the mixtures at 150 rpm for 60 min, followed by filtering the suspensions through Whatman#42 filter papers.

After fumigation, the first set of samples was also divided into two 10-gram portions, which were then extracted similar to the non-fumigated samples. The resulting extracts were stored at 4 °C before analysis.

Total organic C contents in non-fumigated and fumigated extracts were determined using the modified Walkley Black method (Brookes et al., 1985; Vance, Brookes & Jenkinson, 1987). Microbial biomass C was then calculated as follows:

(2) \begin{eqnarray*}\mathrm{MBC}={\mathrm{K}}_{\mathrm{EC}}\ast 2.64\end{eqnarray*}

where K_EC_ is the difference in total organic C contents between the fumigated and non-fumigated samples and 2.64 is the proportionality factor for biomass C released by the fumigation extraction method.

Total N contents in the non-fumigated and fumigated extracts were analyzed following the colorimetric method and MBN was calculated as follows (Brookes, Powlson & Jenkinson, 1982; Anderson & Ingram, 1994):

(3) \begin{eqnarray*}\mathrm{MBN}={\mathrm{F}}_{\mathrm{N}}/0.54\end{eqnarray*}

where *F*_N_ is the difference between total N contents of fumigated and non-fumigated samples and 0.54 is the fractions of biomass N released by fumigation extraction procedure.

Inorganic P concentrations in NaHCO_3_ extracts were ascertained using the ammonium molybdate-ascorbic acid method (Murphy & Riley, 1962). The calculation of microbial biomass P (MBP) involved determining inorganic P contents of fumigated and non-fumigated extracts. Microbial biomass P was calculated as the difference between P contents of fumigated and non-fumigated samples. Additionally, the inorganic P contents of the non-fumigated samples were regarded as the soil P contents.

Ten grams moist soil samples were extracted with a 40-mL 2.0 M KCl solution to analyze soil ammonium-N (NH_4_-N) and nitrate-N (NO_3_-N) contents. The NH_4_-N concentration in the extracts was determined using the sodium dichloroisocyanurate color reagent method, measuring absorbance at 660 nm on a spectrophotometer (Kandeler & Gerber, 1988). For NO_3_-N content analysis, extracts were treated with vanadium(III) for NO_3_-N reduction followed by color development through the Griess reaction, and the absorbance was measured at 540 nm on a spectrophotometer (Miranda et al., 2017). The combined concentration of NH_4_-N and NO_3_-N was considered as the mineral N contents.

### Soil enzymatic activities

#### Dehydrogenase

The dehydrogenase enzymatic activity in soil samples was measured by treating the first set of five g moist soil samples with 5 ml of 2% triphenyl-tetrazolium chloride (TTC) substrate solution (Alef & Nannipieri, 1995). The second control set of samples was mixed with 5 ml of 0.1M tris buffer and the test tubes were placed in an incubator for 16 h at 25 °C. Twenty-five milliliter acetone was added to both test tubes to extract the triphenyl formazan (TPF) contents, and the test tubes were placed on an orbital shaker at 150 rpm for 2 h in the dark. The mixtures were filtered in the semi dark room and absorbance of the extracts was measured photometrically along with calibration standards at 546 nm within an hour. Concentrations of TPF in the filtrates were calculated from the calibration-curve and expressed as µg TPF g^−1^ soil 16 h^−1^.

#### β-Glucosidase

The activity of β-glucosidase in soil samples was quantified using colorimetric method adopted from Dick, Breakwell & Turco (1997). One-gram moist soil samples were treated with 0.2 mL toluene and left to react for 15 mins. After reaction time, one set of samples was mixed with four mL of modified universal buffer solution (MUB, pH 6.0) whereas the second set of samples treated with four mL MUB and one mL 50 mM p-nitrophenyl-β-D-glucopyranoside (PNG) solution. The mixtures were gently shaken and incubated at 37 °C for 1 h. After 1 h, four mL of 0.5M CaCl_2_ and four mL of tris hydroxymethyl aminomethane (THAM; pH 12.0) was added to all samples and, after mixing, one mL of PNG solution was poured into the control set of samples. At this point, the mixtures were filtered, and their absorbance was measured at 420 nm.

#### Urease

For urease activity analysis, five g moist samples were treated with 2.5 mL urea solution in 100 mL Erlenmeyer flasks, and the mixtures along with the sample blanks were incubated at 37 °C for 2 h. After incubation, 50 mL 2M KCl solution was added to mixtures, they were gently mixed on a rotatory shaker, and extracts were filtered through a Whatman#42 filter paper. The extracts and calibration standards (0, 10, 15, 20 and 25 µg NH_4_-N mL^−1^) were analyzed for NH_4_-N concentrations using the colorimetric method described earlier (Kandeler & Gerber, 1988). Urease activity was expressed as µg N g^−1^ 2 h^−1^.

#### Alkaline phosphomonoesterase

The alkaline phosphomonoesterase activity was quantified following the method of Dick, Turco & Breakwell (2015). Two sets of one-gram moist soil samples were treated with 0.2 mL toluene; first set treated with substrate and the other served as the control. The mixtures were left to react for 15 mins. After reaction time, 4 ml of MUB was added to both soil samples; however, one mL p-nitrophenyl phosphate (PNP) was only mixed with soil replicates containing substrate solution. The test tubes were gently swirled, capped and incubated at 37 °C for 1 h. After incubation, 1 ml 0.5M CaCl_2_ and 4 ml 0.5M NaOH were added to all test tubes, and they were mixed thoroughly before filtration. Calibration standards were also prepared using 0, 20, 40, 60, 80 and 100 µg PNP concentrations. The absorbance of the extracts was measured on a spectrophotometer at 420 nm wavelength, and the alkaline phosphomonoesterase activity was presented as µg PNP g^−1^ soil h^−1^

### Statistical analysis

Analysis of variance (ANOVA) test was applied to find the effects of treatments on experimental parameters and to compare the variations between saline, sodic and saline-sodic soils. Duncan’s multiple range post-hoc test was used to rank the significant differences of values between the treatments and soils. All statistical analyses, presented as mean values in Figures and Tables, were performed with SPSS for Windows Software v.19.

## Results

### Characteristics of soils, WS, FYM and BC

The physicochemical properties of saline, sodic and saline sodic soils used in the study are shown in Table 1. Saline, sodic and saline sodic soil were classified as sandy loam, sandy clay loam and silt loam, respectively. Saline, sodic and saline sodic soils had 3.55, 2.70 and 3.90 g kg^−1^ SOC contents, respectively. The sodium content was highest in saline sodic soil (478.7 meq L^−1^) as compared to saline (8.56 meq L^−1^) and sodic soil (20.16 meq L^−1^). pH of WS was less than FYM and BC whereas EC of BC was the highest (Table 2). The corncob BC had the highest total organic C contents (734 g kg^−1^) and C/N ratio (67.3).

**Table 1:** Physico-chemical characteristics of saline, sodic and saline-sodic soils.

Soil properties	Saline soil	Sodic soil	Saline sodic soil
Physical properties			
Sand (g kg^−1^)	596	520	254
Silt (g kg^−1^)	139	116	536
Clay (g kg^−1^)	265	364	210
Textural class	Sandy loam	Sandy clay loam	Silt loam
Chemical properties			
pH	7.72	8.69	8.43
EC (dS m^−1^)	4.23	3.76	64.2
Soil organic matter (g kg^−1^)	6.11	4.64	6.70
SOC (g kg^−1^ soil)	3.55	2.70	3.90
Total N (mg kg^−1^ soil)	305.51	232.0	–
Available P (mg kg^−1^)	6.59	5.86	–
Available K (mg kg^−1^)	146.52	148.76	39.5
Na^+^ content (meq L^−1^)	8.56	20.16	478.7
Ca^2+^ + Mg^2+^ content (meq L^−1^)	2.43	2.94	36.23
HCO_3_^−^ (meq L^−1^)	13.45	38.54	5.25
CO_3_^2−^ (meq L^−1^)	3.65	24.35	–

**Table 2:** Physical and chemical characteristics of wheat straw, biochar and FYM.

Physicochemical characteristics	Wheat straw	Biochar	FYM
pH_1:10_	6.54	7.18	8.11
EC_1:10_ (dS m^−1^)	1.70	5.64	1.34
Total Organic C (g kg^−1^)	416	734	236
Total N (g kg^−1^)	6.41	10.9	12.6
C/N ratio	64.9	67.3	18.7

### Effects on soil pH and EC

Soil pH generally decreased in response to sole and combined additions of BC, FYM and WS (Table 3). In saline soil, a significant decrease in soil pH was found in the BC and FYM treatments in comparison to control. In sodic soil, the lowest soil pH was found in WS and BC plus FYM treatments. However, the difference between the treatments was not significant. In saline sodic soil, pH declined significantly in all treatments except for BC. The lowest pH of 8.31 was observed in WS treated saline-sodic soil.

**Table 3:** Effects of biochar applied alone or with FYM and wheat straw on soil pH and EC of saline, sodic and saline-sodic soils.

Treatments	pH			EC (dS m^−1^)		
	Saline	Sodic	Saline-sodic	Saline	Sodic	Saline-sodic
Control	8.30 ± 0.01 b	8.70 ± 0.02 b	8.63 ± 0.07 c	3.09 ± 0.12 ab	7.06 ± 0.13 ab	5.21 ± 0.04 a
2% BC	8.17 ± 0.03 a	8.53 ± 0.03 ab	8.63 ± 0.03 c	3.14 ± 0.13 ab	8.01 ± 0.73 b	5.18 ± 0.07 a
2% FYM	8.17 ± 0.03 a	8.40 ± 0.12 a	8.37 ± 0.03 ab	3.31 ± 0.06 b	7.30 ± 0.11 ab	5.29 ± 0.06 a
2% WS	8.20 ± 0.06 ab	8.37 ± 0.07 a	8.31 ± 0.11 a	2.89 ± 0.09 a	6.96 ± 0.36 ab	5.13 ± 0.03 a
1% BC+1% FYM	8.27 ± 0.03 ab	8.37 ± 0.09 a	8.43 ± 0.03 b	2.95 ± 0.04 a	7.82 ± 0.47 b	5.17 ± 0.08 a
1% BC+1% WS	8.23 ± 0.03 ab	8.47 ± 0.03 a	8.37 ± 0.03 ab	3.03 ± 0.04 ab	6.48 ± 0.21 a	5.17 ± 0.01 a

**Notes.**

Values are means of three replicates followed by ± standard error of means (*n* = 3). For each parameter and for each soil in a column, values with different letters show significant differences between means values of treatments at *P* < 0.05. BC, Biochar; FYM, Farmyard manure; WS, Wheat straw.

In saline soil, a significant decrease in EC was noted in WS and BC plus FYM treatments as compared to control (Table 3). Similarly, reduction in EC was observed in all treatments applied to sodic soil except BC which resulted in higher EC than control. The lowest soil EC was recorded for the BC plus WS treatment in sodic soil. However, EC of saline-sodic soil did not show significant variations among the treatments, and the values ranged from 5.13 dS m^−1^ for the WS treatment to 5.29 dS m^−1^ for FYM (Table 3).

### Carbon mineralization and CO_2_ efflux

**Figure 1:**
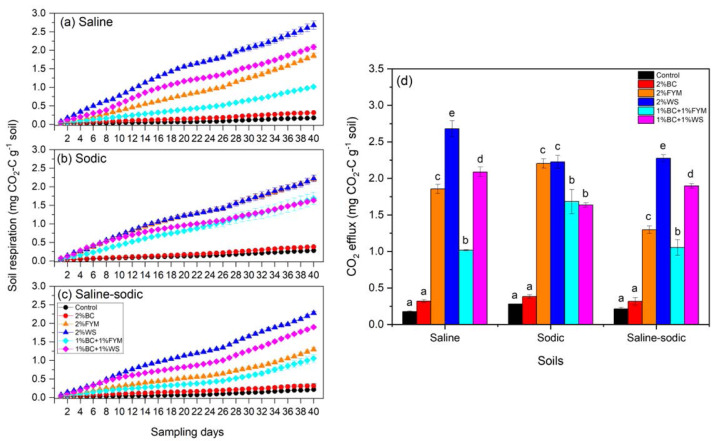
Effects of BC applied alone or with FYM and WS on (A–C) cumulative soil respiration and (D) CO_2_ efflux of saline, sodic and saline-sodic soils. Values are means of three replicates followed and contain ± standard error of means (*n* = 3). In (D), for each soil, bars with different letters show significant differences between the treatments at *P* < 0.05. BC, Biochar; FYM, Farmyard manure; WS, Wheat straw.

Addition of BC with WS and FYM generally reduced C mineralization compared to the WS and FYM only treatments in saline and saline-sodic soils whereas C mineralization was the lowest for the control and BC treatments in all soils (Fig. 1). Moreover, on average, C mineralization values in BC were 1.75 times higher than control. For the saline soil, the highest soil respiration was observed in the WS treatment followed by BC+WS and FYM (Fig. 1A). The combined application of BC with WS and FYM resulted in consistently less soil C mineralization compared to the individual WS and FYM treatments. However, similar C mineralization values were observed when sodic soil received WS and FYM with and without BC (Fig. 1B). Trends in the effects of treatments on soil C mineralization in saline-sodic soil were similar to those observed in the saline soil which followed order: WS > BC+WS > FYM > BC+FYM > BC > ontrol (Fig. 1C). However, the difference between the FYM and BC+FYM treatments were much smaller.

When soil respiration and C mineralization data were presented as CO_2_ efflux, the variations between the treatments and soils became much more plausible (Fig. 1D). Applying BC with FYM and WS significantly reduced CO_2_-efflux compared to the sole FYM and WS treatments. Values of CO_2_-efflux in the control and BC treatments were 8–15 folds less than the FYM, WS, BC+FYM and BC+WS treatments.

### Effects on microbial biomass

**Figure 2:**
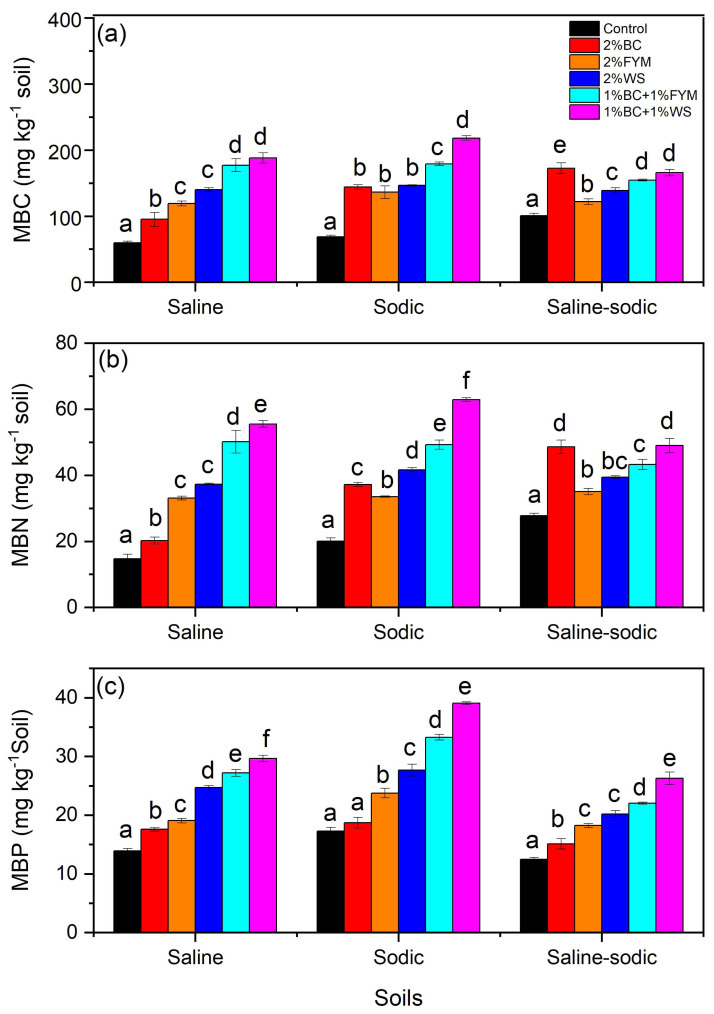
Effects of BC applied alone or with FYM and WS on (A) MBC, (B) MBN and (C) MBP contents of saline, sodic and saline-sodic soils. Values are means of three replicates followed and contain ±standard error of means (*n* = 3). Bars with different letters show significant differences between the treatments at *P* < 0.05. BC, Biochar; FYM, Farmyard manure; WS, Wheat straw; MBC, Microbial biomass carbon; MBN, Microbial biomass nitrogen; MBP, Microbial biomass phosphorus.

In saline soil, MBC was significantly higher after application of BC with WS and FYM as compared to their sole treatments (Fig. 2A). However, the WS, FYM and BC treatments also significantly increased MBC as compared to control. All treatments resulted in significantly higher MBC than control in sodic soil, and the highest MBC value was found under the BC+WS treatment (Fig. 2A). Unlike saline soil, MBC values between BC, FYM and WS did not show significant variations. In contrast to saline and sodic soils, BC resulted in significantly the highest MBC value than other treatments in saline-sodic soil (Fig. 2A). Moreover, the co-application of BC with WS and FYM significantly enhanced MBC in saline-sodic compared to the WS and FYM only treatments (Fig. 2A).

In the saline and sodic soils, MBN followed a pattern of treatments similar to MBC, and significantly the highest MBN contents were found under the BC+WS treatment (Fig. 2B). For saline and sodic soils, MBN significantly increased in all treatments compared to control. However, contrary to saline soil, BC resulted in significantly higher MBN than FYM in sodic soil. The BC only and BC+WS treatments increased MBN significantly than other treatments in saline-sodic soil, and the variations among FYM, WS and BC+WS were less pronounced (Fig. 2B).

Effects of treatments were more consistent on MBP in all soils; however, MBP values in saline-sodic soil under each treatment were less than saline and sodic soils (Fig. 2C). For all soils, the BC+WS treatment always showed the significantly highest MBP values, and the general order of MBP was BC+WS > BC+FYM > WS > FYM > BC > control (Fig. 2C).

### Changes in extractable and total soil organic C

**Figure 3:**
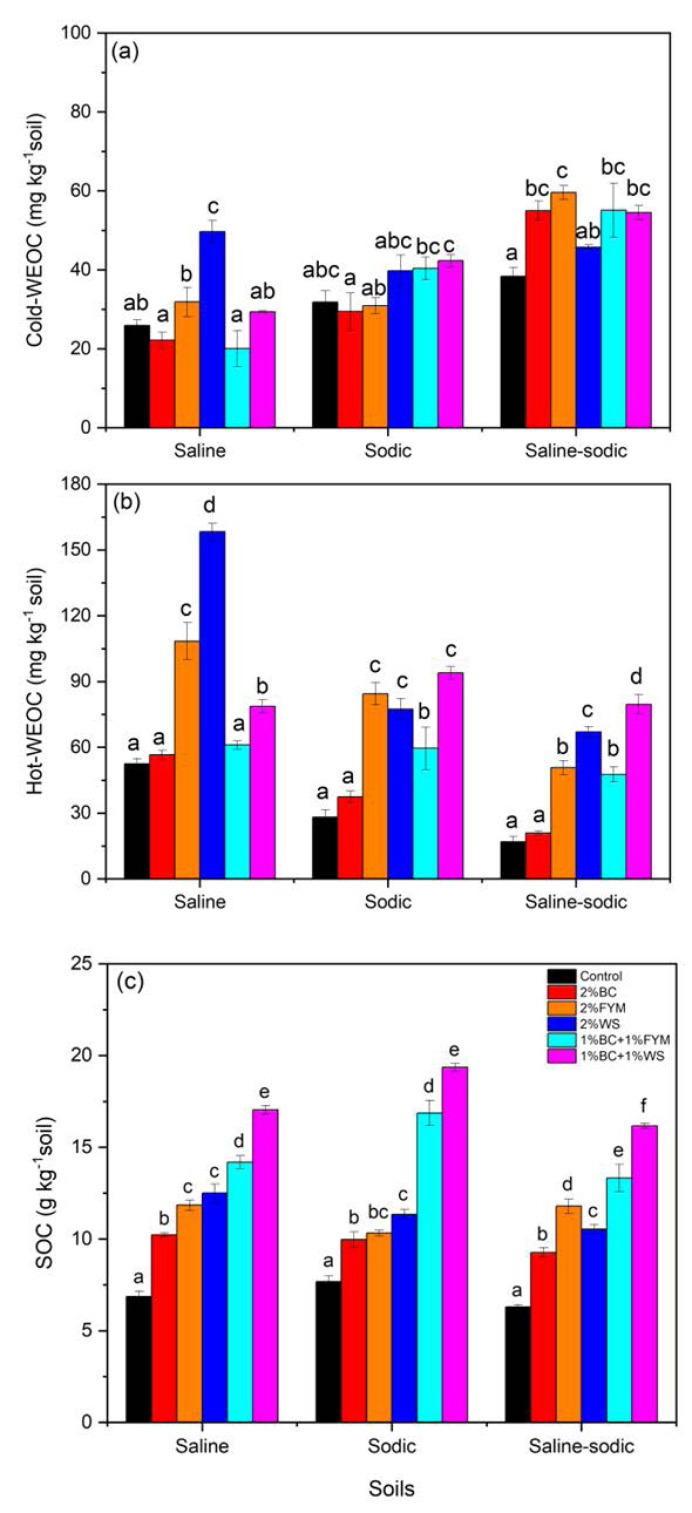
Effects of BC applied alone or with FYM and WS on (A) cold-WEOC, (B) hot-WEOC and (C) SOC contents of saline, sodic and saline-sodic soils. Values are means of three replicates followed and contain ±standard error of means (*n* = 3). Bars with different letters show significant differences between the treatments at *P* < 0.05. BC, Biochar; FYM, Farmyard manure; WS, Wheat straw; WEOC, Water extractable organic carbon; SOC, Soil organic carbon.

In saline soil, the WS treatment significantly increased cold-WEOC contents in comparison to all other treatments (Fig. 3A). Despite the higher cold-WEOC contents in the WS, BC+WS and BC+FYM treatments, sharp and significant contrasts did not occur between the treatments for sodic soil. Saline-sodic soil generally exhibited higher cold-WEOC contents under each treatment compared to saline and sodic soils (Fig. 3A). In saline-sodic soil, FYM had the highest cold-WEOC contents, but it was significantly higher than the control and WS treatments only.

The WS treatment had the highest hot-WEOC contents in saline soil followed by FYM and BC+WS treatments, respectively (Fig. 3B). In sodic soil, BC, FYM and BC+WS resulted in significantly higher hot-WEOC contents than the control, BC and BC+FYM treatments. Similar treatments effects were noted for saline-sodic soil; however, the hot-WEOC contents were less under each treatment than those observed for the saline and sodic soils (Fig. 3B).

The increase in SOC followed a similar pattern in saline, sodic and saline-sodic soils (Fig. 3C). The highest SOC contents were found in the BC+WS treatment followed by BC+FYM. Additions of BC alone and with FYM and WS always resulted in significantly higher SOC than control in all soils. In sodic soil, BC with WS and FYM resulted in much sharper significant increase of SOC than other treatments (Fig. 3C).

### Effects on mineral N and P contents

**Figure 4:**
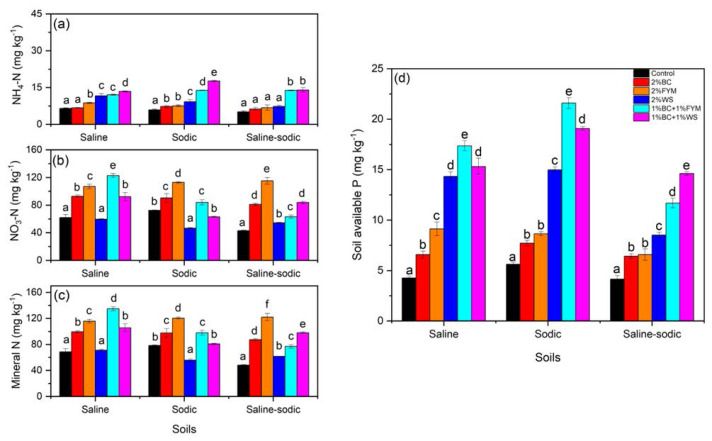
Effects of BC applied alone or with FYM and WS on (A) NH_4_-N (B) NO_3_-N, (C) mineral N and (D) soil available P contents of saline, sodic and saline-sodic soils. Values are means of three replicates followed and contain ±standard error of means (*n* = 3). Bars with different letters show significant differences between the treatments at *P* < 0.05. BC, Biochar; FYM, Farmyard manure; WS, Wheat straw.

Application of FYM, WS, BC+FYM and BC+WS significantly enhanced NH_4_-N contents compared to the control and BC treatments in saline soil (Fig. 4A). Similar effects of treatments were observed for the sodic soil where the highest NH_4_-N content was measured under the BC+WS treatment. However, in saline-sodic soil, BC with FYM and WS resulted in significantly the highest NH_4_-N contents compared to the control, BC, FYM and WS treatments (Fig. 4A). Effects of treatments on soil NO_3_-N contents in saline, sodic and saline-sodic soils revealed the lowest NO_3_-N contents under the WS treatment (Fig. 4B). Saline soil treated with BC showed significantly the highest NO_3_-N contents in contrast to the sodic and saline sodic soils where the highest NO_3_-N contents were found after the FYM treatment. Combining BC with FYM significantly reduced NO_3_-N than the FYM only treatment in sodic and saline-sodic soils but not in saline soil. As NO_3_-N was the dominant N pool than NH_4_-N, and they both collectively represented mineral N contents, hence the treatments effects on mineral N contents followed trends similar to those found for NO_3_-N contents for saline, sodic and saline-sodic soil (Figs. 4B & 4C).

Soil available P contents varied significantly between the treatments and among the soils (Fig. 4D). The WS, BC+FYM and BC+WS treatments resulted in significantly higher soil available P contents compared to control, BC and FYM in all three soils. The highest soil available P contents were found under the BC+FYM treatments in saline and sodic soil. However, in saline-sodic soil, BC+WS resulted in the highest soil available P contents.

### Effects on quantity and quality of DOC

**Figure 5:**
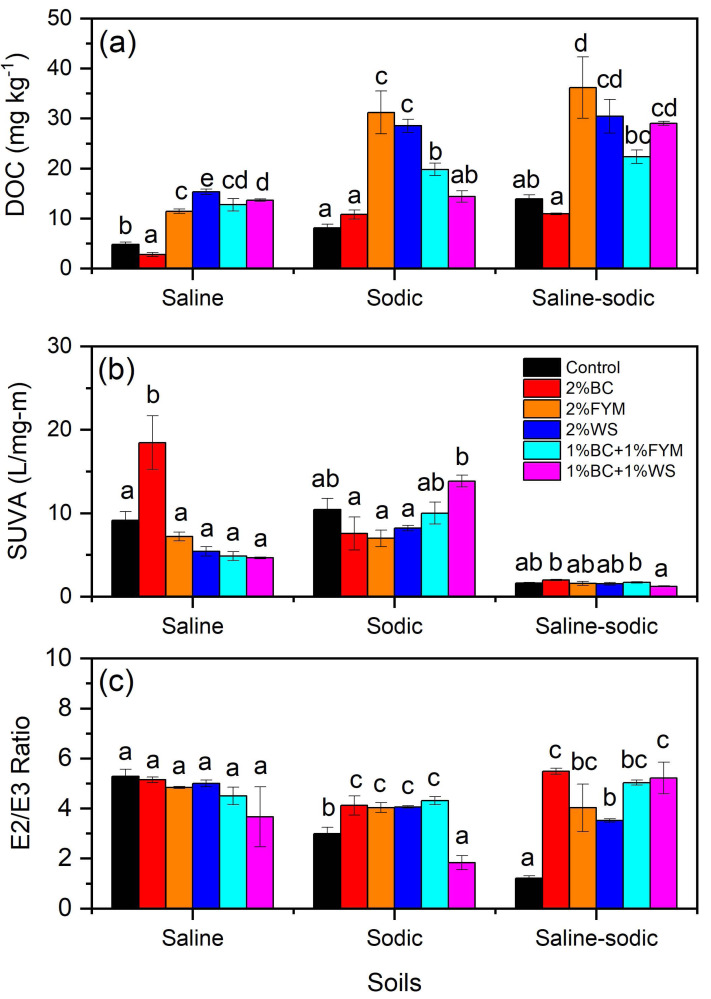
Effects of BC applied alone or with FYM and WS on (A) DOC (B) SUVA and (C) E2/E3 ratio of saline, sodic and saline-sodic soils. Values are means of three replicates followed and contain ± standard error of means (*n* = 3). Bars with different letters show significant differences between the treatments at *P* < 0.05. BC, Biochar; FYM, Farmyard manure; WS, Wheat straw; DOC, Dissolved organic carbon; SUVA, Specific ultraviolet absorbance.

In saline soil, DOC contents were significantly higher in the FYM, WS, BC+FYM and BC+WS treatments than the control and BC treatments, and BC had the lowest DOC contents (Fig. 5A). The FYM and WS treatments significantly increased DOC contents compared to other treatments in sodic soil whereas only the FYM treatment showed significantly the highest DOC contents than the remaining treatments in saline-sodic soil. In saline, sodic and saline-sodic soils, the lowest DOC contents were observed under the control and BC treatments. When compared the effects of FYM on DOC contents between the soils, DOC was >2-folds higher in sodic and saline-sodic soils than saline soil (Fig. 5A).

Saline, sodic and saline-sodic soils exhibited contrasting variations in specific ultraviolet absorbance (SUVA) values (Fig. 5B). In saline soil, the BC treatment demonstrated the significantly higher SUVA value. Moreover, the rest of the treatments were neither significantly different from each other nor from control. In sodic soil, despite the higher SUVA values under the control and BS+WS treatments, the trend in treatment effects was non-significant and lacked consistency. Unlike saline and sodic soils, SUVA values were much less under each treatment in saline-sodic soil without any noticeable variations (Fig. 5B).

In saline soil, none of the treatments exhibited significantly different values of E2/E3 ratio from control (Fig. 5C). However, in sodic soil, the BC, FYM, WS and BC+FYM treatments showed significantly higher E2/E3 ratio than control and BC+WS whereas significantly the lowest E2/E3 ratio value was observed under the BC+WS treatment. The control treatment had significantly the lowest E2/E3 ratio compared to the other treatments in saline sodic soil. Moreover, E2/E3 ratio values in the BC and BC+WS treatments were similar but significantly higher than control and WS (Fig. 5C).

### Effects on soil enzymatic activities

**Figure 6:**
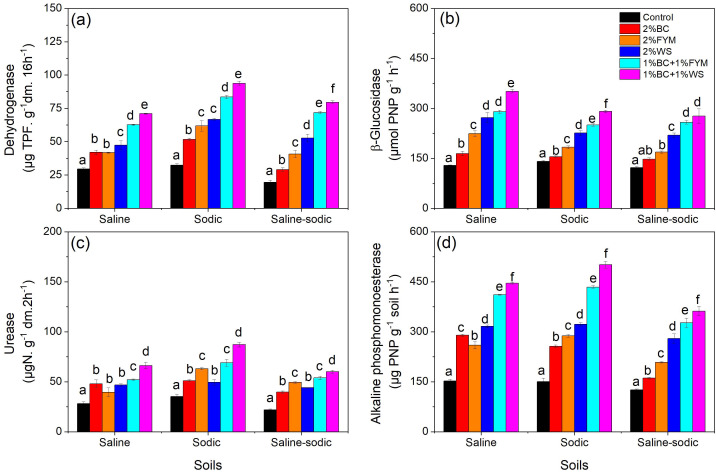
Effects of BC applied alone or with FYM and WS on (A) dehydrogenase (B) β-glucosidase (C) urease and (D) alkaline phosphomonoesterase activities of saline, sodic and saline-sodic soils. Values are means of three replicates followed and contain ± standard error of means (*n* = 3). Bars with different letters show significant differences between the treatments at *P* < 0.05. BC, Biochar; FYM, Farmyard manure; WS, Wheat straw.

The soil dehydrogenase activity significantly increased in all three soils in response to treatments, but the highest dehydrogenase activity was observed in sodic soil (Fig. 6A). The highest and lowest dehydrogenase activity was noted under the BC+WS and control treatments, respectively in saline, sodic and saline-sodic soils. Increase in dehydrogenase activity values followed a similar order in all three soils: BC+WS > BC+FYM > WS > FYM > BC > control.

The β-glucosidase activity significantly increased under all treatments in saline, sodic and saline sodic soils but the highest β-glucosidase activity was observed in saline particularly under the BC, FYM, WS, BC+FYM and BC+WS treatments (Fig. 6B). The effects of treatments on β-glucosidase activity in three soils followed the order similar to dehydrogenase activity. Moreover, in saline soil, the BC and BC+FYM treatments and, in saline sodic soil, BC+FYM and BC+WS treatments were not significantly difference from each other.

The treatments induced significant increase in urease activity of saline, sodic and saline sodic soil compared to control but the urease activity was generally the highest in sodic soil (Fig. 6C). In saline, sodic and saline-sodic soils, the urease activity was significantly the highest in the BC+WS treatment compared to all other treatments. In saline, sodic and saline-sodic soils, the BC, FYM and WS treatments alone and in-combination significantly enhanced alkaline phosphomonoesterase activity compared to control, and the highest alkaline phosphomonoesterase activity was always observed under the BC+WS treatment in all soils (Fig. 6D). All treatments resulted in significantly higher alkaline phosphomonoesterase activity than control in all soils. In saline and sodic soils, the increase in the alkaline phosphomonoesterase activity under the BC+FYM and BC+WS treatments was much higher than the other treatments. However, unlike saline soil, the increase in alkaline phosphomonoesterase activity in the sodic and saline sodic soil followed order: r BC+WS > BC+FYM > WS > FYM > BC > control.

## Discussion

### Soil pH and EC

In this study, a decrease in pH of saline, sodic and saline-sodic soil was observed in sole and combined application of BC, FYM and WS which could be attributed to increased microbial activity leading to accumulation of organic and inorganic acids and caused a decline in soil pH. This observation is consistent with the findings of Yang et al. (2018) who also detected a decline in pH of saline soil following the application of organic amendments. In saline and sodic soils, when applied alone, BC and FYM enhanced EC but combining BC with FYM and WS reduced EC; however, treatment effects on EC in saline-sodic soil were non-significant and non-consistent. Similar to our findings, Jatav et al. (2018) also reported that rice husk biochar did not result in any significant changes in soil EC. Use of alkaline BC in alkaline soils is generally critically viewed because of its potential ability to enhance soil pH that could suppress beneficial microbial activities and immobilize nutrients causing nutrient deficiencies, retarded plant growth and reduce crop yields (Jeffery et al., 2017; Dai et al., 2020). However, in our study, pH of BC was slightly alkaline (7.18) which was added to alkaline calcareous saline, sodic and saline-sodic soils with much higher pH values of pH 7.72, 8.69 and 8.43, respectively. Adding BC alone or with FYM and WS reduced but never enhanced soil pH suggesting potential benefits of BC and BC combined with FYM and WS to counter-balance pH and promoting microbial activity and nutrient availability in our study. A recent meta-analysis by Wang et al. (2024) also reported that BC ameliorated salinity stress in salt-affected soils without any pronounced effects on soil pH.

### Carbon mineralization and microbial activity

The application of organic amendments such as crop residues and manures stimulates soil C mineralization due to higher availability of labile C (Dodor et al., 2018). We found that the sole application of WS and FYM significantly enhanced microbial activity and microbial biomass (MBC, MBN and MBP), in saline, sodic and saline-sodic soil probably due to higher availability of easily degradable labile C fractions, evident from higher hot and cold WEOC contents in the WS and FYM treatments. The sole application of WS showed higher soil respiration and CO_2_ efflux than sole application of FYM. Despite high C:N ratio of WS, it also had high WEOC contents suggesting presence of larger labile C pool specifically associated with cellulose and hemicelluloses which could have been mineralized by soil microorganisms (Yazdanpanah, 2016). However, in contrast, the lower C mineralization and CO_2_ efflux in FYM than WS suggests the presence of larger amount of recalcitrant C linked to hemicelluloses and lignin which are less preferred for decomposition by microbes (Elmajdoub & Marschner, 2013; Chahal, Choudhary & Mavi, 2018).

Application of BC alone resulted in slightly higher C mineralization than control, but the differences were non-significant. However, higher C mineralization was observed when BC was combined with WS and FYM. In addition, the combined application of WS and FYM with BC resulted in significantly higher MBC, MBN and MBP contents indicating synergistic effects of BC on microbial activity and nutrient acquisition efficiency when combined with WS and FYM. Higher microbial growth under the combined addition of BC, WS and FYM also provides evidence for the presence of co-metabolism and favorable niche that supports microbial activity. However, these mechanisms could not be fully supported from our experimental set which did not allow to quantify partitioning between C respired from BC and added organic matter (WS and FYM). Lower CO_2_ efflux at the expense of higher microbial biomass in the BC+FYM and BC+WS treatments than sole FYM and WS could suggest a BC-induced negative priming effect which apparently enhanced microbial CNP utilization efficiency (Riaz et al., 2017). In saline soil, Manasa et al. (2020) also found negative priming effects on native SOC and suggested that BC along with digestate amendment could be a promising approach to reclaim saline soils on long-term basis. Addition of organic amendments to soil increases microbial activity and biomass by providing readily decomposable C and essential nutrients (Tripathi et al., 2006). In this study, the combined application of BC with WS and FYM significantly increased MBC than their individual applications, particularly in saline and sodic soils. Lehmann et al. (2011) suggested that biochar influenced microbial populations through various mechanisms, including creation of more favorable soil physico-chemical environment and microhabitats that possibly offer additional opportunities for microbial growth and development. That could explain the higher MBC contents in soils received combined application of BC with WS and FYM than those treated with BC alone. These results are supported by the findings of Cardelli et al. (2017), who reported increase in soil MBC when compost (vermi-compost & green compost) was applied with BC. Similarly, Liu et al. (2016) noted significant effects on MBC when BC and composted-waste were added together alongside. Moreover, similar to MBC in our BC alone treated soils, Fatima et al. (2021) found higher MBC in BC treated soil that increased with rate of BC application. Similar to our study about saline soils, Chahal, Choudhary & Mavi (2018) & Bhullar, Mavi & Choudhary (2019) found higher microbial activity and nutrient released when BC was applied to saline soils suggesting the role of BC to reduce the impact of salt-stress on microbes. Conversely, Dempster et al. (2011) found a reduction in soil MBC when Eucalyptus-derived BC was co-applied with WS and composted manure or with N fertilizer. Some short-term studies also have indicated that BC did not significantly improve MBC under both the absence (Zavalloni et al., 2011) and presence of salt stress conditions (Chaganti, Crohn & Šimůnek, 2015). These findings suggest that not all BCs could enhance microbial activity and biomass as BC properties heavily depend on feedstock nature and quality, and hence, some BCs could contain toxic substances known to inhibit microbial activity (Zimmerman, Ahn & Gao, 2011; Buss et al., 2015). Moreover, such conflicting reports about the BC effects on microbial activity in the literature also indicate variations in factors such as the BC production conditions, its application rates, experimental settings (greenhouse/field, soil type, and climate), and the conditions of BC (fresh, aged, pre-treated) (Cai et al., 2021).

In addition to MBC contents, addition of BC, FYM and WS alone and in-combination also concurrently increased MBN and MBP contents in saline, sodic and saline-sodic soils in this study. This could have been due to the enhanced microbial activity and resultant higher N and P utilization to meet microbial metabolic demands (Akmal et al., 2019).

### Quantity and quality of SOC

Our study found a significant increase in SOC contents in salt-stressed soils after application of BC with or without WS and FYM, and the highest SOC was observed when BC was combined with WS and FYM. These findings are primarily attributed to the substantial aromatic C content found in BC as the aromatic C is highly stable and supports accumulation of SOC (Chen et al., 2019). Earlier studies have shown that the incorporation of BC to soils triggers a broad spectrum of effects due to its extensive surface area that enhances microbial activity and their adhesion to BC (Elad et al., 2010; Lehmann et al., 2011). Resultantly, these processes lead to an increased polymerization of organic molecules within soil and encourage organic matter formation (Graber et al., 2010). Water extractable organic C is an immediate product of soil organic matter decomposition and acts as the primary source of energy that fuels microbial activity (Feng et al., 2024). In this study, the individual applications of WS and FYM had higher WEOC contents in saline soil than when WS and FYM were applied with BC, but this trend was different for sodic and saline-sodic soils where adding BC with WS resulted in the highest WEOC contents (Amini et al., 2016).

Under higher SOC contents, a possible decline in salt stress could lead to higher microbial activity and microbial biomass that improved soil fertility and functions (Singh, Singh & Singh, 2013). When organic amendments are added to saline soils, they enhance SOC contents and mediate salinity stress to foster microbial functions, nutrient availability, and crop yields (Farooqi et al., 2023). A meta-analysis by Li et al. (2023) provides further evidence that organic amendments including BC significantly enhance SOC, soil functions and crop productivity in saline soils.

Along with WEOC, DOC also represent labile C fractions of SOC and servers as an indicator of SOC lability (Ibrahim et al., 2021). Despite being a relatively smaller component of SOC pools, these labile C fractions support multiple functions within the soil ecosystem (Han et al., 2020). In this study, DOC contents increased when WS and FYM were added alone as well as in combination with BC, and the highest DOC contents occurred under the sole applications of FYM and WS. Addition of organic amendments such as straw and manures stimulate microbial activity, and resultant decomposition raises labile C contents similar to those observed for the WS and FYM treatments in our study which led to higher DOC contents in soil. However, the decline in DOC contents in the BC+FYM and BC+WS treatments in this study probably was attributed to DOC adsorption onto BC surface (Ibrahim et al., 2021). In addition to changes in DOC contents, these interactions of DOC with BC could alter the quality of labile C and thus affect its degradability as evidenced from SUVA values in our study. We found that SUVA, a marker of dissolved organic matter aromaticity, increased under the BC only and BC+WS treatments in saline and sodic soils, respectively which can be associated with monomer polymerization and aromatization (Song et al., 2020). Moreover, compared with control, E2/E3 ratio values were higher in sodic soil in all treatments excluding BC+WS, whereas all treatments in saline-sodic soil enhanced E2/E3 ratio than control indicating at decreased molecular weight of dissolved organic matter in these soils (Lee et al., 2022).

### Soil N and P dynamics

Increased nutrient availability is usually linked with improved soil fertility. In this study, application of BC with WS and FYM significantly enhanced the availability of NH_4_-N, NO_3_-N, mineral N and P contents in saline, sodic and saline-sodic soil which is similar to findings from other studies (*e.g.*, Guo, Liu & Zhang, 2020; Cui et al., 2020). Moreover, BC added with crop residues can facilitate recycling of essential macro and micronutrients, and thus leading to soil enhanced nutrient availability (Singh & Mavi, 2018). This can also be due to adsorption of N and P onto biochar surface that lead to increased total N and P contents in soil (Zhou et al., 2021). Furthermore, BC can stimulate mineralization of N and causing a decline in soil pH which increases P solubility and efficiency (Chen et al., 2021). Increase in soil mineral N contents can also be attributed to substantial amount of N provided by addition of organic amendments such as WS and FYM in this study. Biochar is known to stimulate N availability as well as its retention in soils, thus improving N use efficiency and reducing N loses to environment (Ahmad et al., 2021). Moreover, the findings from limited data suggest that BC also improve N dynamics in saline soils but effects largely varied with nature of BC and extent of salinity (Amini et al., 2016). Although minor in our study, but the decreased soil pH could have positive impacts on P solubility of phosphate whereas the production of organic acids from organic matter decomposition also stimulate P release, ultimately increasing P availability in soil (Lakhdar et al., 2009; Wang et al., 2015). The ability of BC to modify soil characteristics such as calcium-related chemical reactions particularly in alkaline soils could increase P desorption and enhance soil P availability (Subedi et al., 2016). Thus, BC amendments increase soil P not only by direct but also indirect mechanisms including inducing changes in soil physicochemical and biological properties (Zhang et al., 2025).

### Activities of soil extra-cellular enzymes

Soil extra-cellular enzymes are highly responsive to abiotic environmental stresses and valuable indicators of soil health. Soil salinity has been known to cause negative effects on enzymatic activities decreasing microbial biomass and nutrients’ availability (D’Hose et al., 2018). Our study demonstrated that BC applied alone and with WS and FYM significantly increased the activities of dehydrogenase, β-glucosidase, urease and alkaline phosphomonoesterase enzymes in saline, sodic and saline-sodic soils. When BC and other organic amendments are added to the soil, they improve the intricate connection between soil enzymes and soil nutrient availability by improving soil physicochemical properties, and subsequently enhance soil quality (Xian, Wang & Chen, 2015; Liang et al., 2017; Yoo et al., 2018). This generally occurs due to higher microbial mining of labile C substrate originating from the release of organic amendments, and thus raising levels of extra-cellular enzymatic activities (Jia et al., 2017; Abou Jaoude et al., 2020). In our study, the higher dehydrogenase and β-glucosidase activities in response to the treatments involving BC can be related to volatile compounds present in BC at lower temperature (<500 °C), BC tends to have higher volatile matter which could accelerate these enzymatic-activities in sandy loam soil, including activities of enzymes (Ameloot et al., 2013).

The increased urease activity might be driven by microorganisms that expedite N mineralization due to the accelerated microbial activity to counterbalance the elevated C/N ratios following the addition of BC (Tian et al., 2016). However, the constraints of labile C deficiency in BC can be mitigated and supplemented by adding BC with other organic amendments to augment enzymatic activity and nutrient availability (Bruun et al., 2011; Joseph et al., 2013). This is supported by the synergistic improvements recorded in soil available nutrients and enzymatic activities resulting from the application of BC with WS and FYM rather than its individual application in this study. It could be assumed that the combined application of BC with WS and FYM relieved sodium toxicity and created more favorable conditions for microbial growth and functions (Agegnehu, Srivastava & Bird, 2017; Qu et al., 2020). Thus, BC can increase activities of C, N and P acquisition enzymes particularly in degraded soils with low nutrient availability such as saline soils by promoting microbial activity, SOC contents and altering microbial community structure and functions (Pokharel, Ma & Chang, 2020; Jaffar et al., 2024).

### Conclusion

Our study showed that, compared to control, BC with WS and FYM pronouncedly improved microbial activity and biomass (four times higher), SOC (2–3 times higher), soil enzymatic activities (4–5 folds higher) and soil N (46–63% higher) and P (4 times higher) availability. Higher microbial biomass at the expense of lesser C mineralization after addition of BC with FYM and WS suggested increase in microbial C utilization efficiency and shift in their metabolic functions. However, the nature and extent of these interactive effects strongly depended on the degree of soil salinity. We also noticed pronounced variations in SOC quantity and quality, indicated by changes in labile C characteristics, when BC was applied with WS and FYM suggesting implications of SOC stability and decomposition. Our study also showed that BC could improve soil physicochemical and biochemical functions and promote C sequestration in saline soils leading to positive effects on soil quality. Moreover, adding BC with organic matter and nutrient rich WS and FYM was more useful in improving microbial properties and soil multifunctionality under salinity stress.

## Supplemental Information

10.7717/peerj.21100/supp-1 Supplemental Information 1 Raw data

## Additional Information and Declarations

### Data Availability

The following information was supplied regarding data availability: The raw data is available in the Supplemental File.
